# Dr. Levi Colby Taylor

**Published:** 1922-10

**Authors:** 


### DR. LEVI COLBY TAYLOR

Dr. Levi Colby Taylor died February 8, 1922, in Hartford, Conn., in his eighty-first year. Born in New Hampshire in 1841.

He began the study of dentistry in Springfield, Vt., as an apprentice, where he studied and worked for several years. In 1868 he began practice in Holyoke, Mass. In 1875 he moved to Hartford, Conn., and was associated in practice with Dr. John M. Riggs for several years.

Dr. Taylor was a man of sterling character, and devoted himself to the strictest ethical ideals. Though not having had the advantages of a college training, he always studied and was greatly interested in dentistry and its professional activities. He was greatly interested in the treatment of pyorrhea and associated departments of the profession. He became intensely interested in prophylaxis and did much to encourage the work Dr. D. D. Smith, of Philadelphia, was so faithfully inducing the profession to enter upon. For several years Dr.

Taylor gave lectures in the New York College of Dental and Oral Surgery, giving special attention to oral prophylaxis.

Dr. Taylor was a constant attendant at all the dental societies of his vicinity. He wrote much on his special topics and on general operative dentistry. He was highly appreciated for his thoughtful considerations of all technical dental topics.

He has had a long and profitable life, which he has given unqualifiedly to his beloved profession. He has made for himself a name and reputation that few can claim. He has served humanity in the way that it seemed to him highest and best, and many will regret his passing away. Those who were privileged to have known him will never forget his earnestness and devoted interest to the profession.

We are printing in these pages a complete program of the Ohio State Dental Society. We trust the readers of the Dental Register will not feel that this is an unwarranted infringement on their time and space. This is an unusual occasion, and we justify the inovation because of its professional importance on this occasion.

